# Total antioxidant capacity of saliva and dental caries

**DOI:** 10.4317/medoral.18762

**Published:** 2013-03-25

**Authors:** Fatemeh Ahmadi-Motamayel, Mohammad T. Goodarzi, Seyedeh S. Hendi, Shahin Kasraei, Abbas Moghimbeigi

**Affiliations:** 1 Research Center for Molecular Medicine, Hamadan University of Medical Sciences, Hamadan Iran; 2 Dental School Hamadan University of Medical Sciences, Hamadan Iran; 3 Dental Research Center Hamadan University of Medical Science, Hamadan Iran; 4 Department of Biostatistic School of Public Health, Hamadan University of Medical Sciences, Hamadan Iran

## Abstract

Objective: Dental caries is one of the most common infectious diseases worldwide. Saliva has many functions in the oral cavity and is the first line defense against dental caries. Oxidative stress can affect initiation and progression of many inflammatory and infectious diseases such as dental caries. Thus the aim of this study was to evaluate the relationship between total antioxidant capacity (TAC) of saliva and dental caries. Study Design: 100 healthy high school students (50 female and 50 male) with age range of 15 -17 years were randomly selected, divided to four groups. Unstimulated whole saliva specimens were collected at the morning. TAC of saliva was evaluated by spectrophotometric assay. Statistical comparisons were performed using Student’s t-test, by SPSS 13. Results: The level of TAC was significantly higher in the saliva of caries active group relative to the caries free subjects. Statistical analysis for male and female groups showed a statistically significant reduction of TAC level in female group. Conclusion: TAC was higher in caries active group. Thus this result showed that total antioxidant capacity may influence in dental caries and activity can be measured by salivary factors and this may be helpful in preventive dentistry.

** Key words:**Dental caries, saliva, total antioxidant capacity.

## Introduction

Saliva is a complex fluid in the oral cavity, composed of a mixture of secretary products from the major and minor salivary glands (1). Saliva has multifunctional roles in the oral cavity (2), and is very important for the maintaining of oral health (3). Thus the saliva research field is rapidly advancing.

About 99% of saliva is water (1,4) The remaining 1% is complex of organic and inorganic molecules, such as electrolytes, mucins, antiseptic substances, immunoglobulin, proteins and various enzymes (1,5). Although the main component of saliva is water, it play key roles in lubrication, mastication, taste perception, prevention of oral infection and dental caries (1,6,7).

Saliva has various defense mechanisms such as immunological and enzymatic defense systems against bacteria, viruses, fungi, protection of mucosa and promotes its healing (8,9). One of the important defense mechanism is antioxidants system (8). Antioxidants have many health benefits that made their evaluation in disease process very popular (10).

Caries is one of the most common oral health problems and its prevention is one of the most important strategies in many countries. The levels of antioxidants could be changed in response to an infection, inflammation or disease (11). For example saliva peroxidase controls oral bacteria which lead to dental caries and periodontal diseases (12).

The salivary antioxidant system is made of various enzymes (peroxidase, catalase, super oxide dismutase, gluthation peroxidase) and small molecules( uric acid, vitamin E,C) (13,14). Amitha M investigated the total antioxidant capacity (TAC) of saliva and its relation with early childhood caries and rampant caries. The results indicated that TAC of saliva was increased in children with caries (11).

Also Tulunglu showed that total protein and total antioxidant level of saliva were increased with caries activity (12).

Studies showed that females had higher protein concentrations than males (15).

However, very little studies have been discussed about the TCA of saliva with dental caries. Therefore, the aim of this study was to evaluate the relationship between the TAC and dental caries.

## Material and Methods

100 healthy high school students (50 female and 50 male) with age range of 15 -17 years were randomly selected and participated in this study. Written informed consent was obtained from all subjects. And confirmation from ethics committee of Hamadan University of Medical Sciences was obtained. Exclusion criteria were systemic disease, medication, smoking, periodontal disease and poor oral hygiene. subjects taking. Subjects were divided to four groups as fallows:

Caries free female (CF), caries active females (CA), caries free males, caries active males, each group consisted 25 subjects.

-Saliva sampling

Un-stimulated whole saliva specimens were collected at the morning, and it was asked from all selected students that brush their teeth and do not use any oral stimulation such as eating and drinking for 90 min prior to collection (16). Students were in sitting and anterior head protrusion position. Whole saliva samples were obtained by expectorating into polypropylene tubes within 5 minutes The saliva sample were first weighed and reweighed again then immediately put on to ice and stored in 4oC and transferred to the laboratory up to 20 minutes and kept at -80oC until the analysis.

-Clinical examination

All clinical examination was carried out by single examiner. Caries detection was based only on clinical caries that observed with dental mirror and explorer and radiographic examination was not performed. CA group were selected within the subjects that had at least five clinical caries surface. And CF group were students that did not have any caries and filling and sign and symptom of sensitivity of teeth (DMFT=0). All selected group had same age range.

-Sialochemical analysis

TAC measurement on saliva samples was carried out using the antioxidant assay kit (Cat #709001; Cayman Chemical). The reaction was based on the ability of aqueous and lipid antioxidants to inhibit the oxidation of the 2,2’-Azino-di-[3-ethylbenzthiazoline sulphonate] (ABTS) to ABTS‏+. The capacity of the antioxidants to prevent ABTS oxidation was then compared with that of standard Trolox, a water soluble tocopherol analogue. Absorbance was measured at 405 nm according to the kit manufacturer`s instructions using Tecan Sunrise microplate reader (Tecan, Austria).

-Statistical analysis

Data were analyzed with SPSS 13.Statistical comparisons were performed using Student’s t-test. The values are expressed as mean +- SD. A p value of <0.05 was considered statistically significant.

## Results

The results showed that CA group had higher TAC than CF group ( p< 0.001). TAC in CA females was higher than those of CF females but the difference was not statistically significant (p<0.84). CA males had statistically significant higher TAC than CF male (p=0.000). Statistical analysis for male and females groups showed a statistically significant lower value of TAC in female group ( p < 0.001). CA females had lower TAC than CA males (p= 0.000) also similar result was observed in CF group indi-cating lower TAC in CF female compared to CF males but the difference was not statistically significant (p< 0.710). The mean and standard deviations of each group is shown in  Table 1. TAC of all 100 students were 45/58 ±14/81.

**Table 1:**
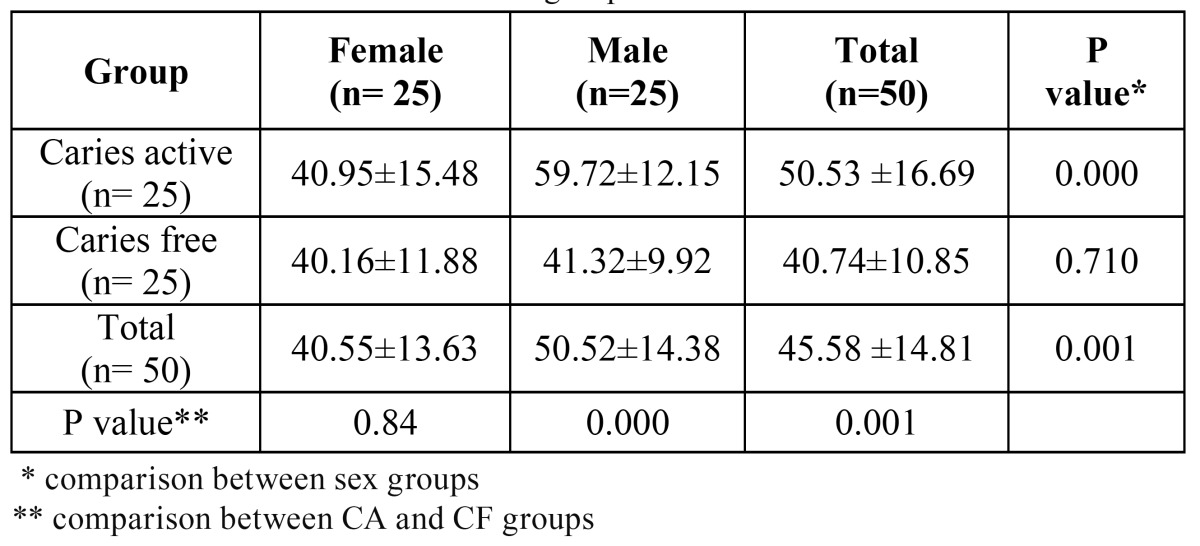
TAC level in different studied groups.

## Discussion

Saliva antioxidant defense mechanisms seem to be very important. There are very few little studies discussed about relationship between TAC of saliva and dental caries (11,12). Thus we evaluated the TAC of saliva in caries active females, CA males, CF females and CF males. We aimed to find the relation between caries and TAC of saliva in males and females.

Our result was similar to results of other studies. Hegde AM investigated the TAC of saliva and its relation with early childhood caries and rampant caries. They indicated that TAC of saliva was increased in children with caries (11). Tulunglu showed that total protein and TAC of saliva were increased with caries activity (12). Preethi B.P in a study about evaluation of physicochemical properties of saliva and TAC also reported higher TAC in CA group (17). Dodwad R and coworkers studied TAC in two CA and CF groups of children and showed increased TAC in CA group (18). Kumar reported significant evaluation of TAC of saliva in children with severe early childhood caries (19).

Although other studies showed that parotid saliva is the major source of the antioxidant in saliva (8) In this study we examined whole saliva that is a complex mixture of oral fluids from salivary glands secretions in addition to several constituents of non-salivary origin which is present in the mouth (1,20,21).

Also un-stimulated saliva sample were taken because other studies showed that TAC was higher in unstimulated saliva (11,12,22).

In this study TAC of saliva was evaluated because any antioxidant work together (23), other studies suggested that TAC evaluation was statistically significant than individual antioxidants (24) the measurement of any individual antioxidant may be difficult and expensive, TAC shows the action of all non-enzymatic antioxidant (25) in addition this results may be a guide for future researcher to work in individual antioxidant activity in caries.

In according to other studies our result also showed that TAC, an important factor of saliva increased in students with caries (13,14).

CA group had higher TAC than CF group and the difference was statistically significant. Similar result was observed in comparison of CA female with CF female and CA male with CF male. Female group had statistically significant lower TAC than male group also similar result was observed in comparison of CA female with CA male and CF female with CF male.

Thus it can be concluded that this TAC elevation can be related to more oxidative stress due to caries in CA group than CF group. As well as TAC may be increase with increasing salivary protein level because salivary proteins interfere with bacterial colonization when bacterial load were elevated (24). Also one of the salivary antioxidants functions are control of oral bacteria that form dental plaque and lead to dental caries and periodontal disease. Because TAC can be elevate in poor oral hygiene and periodontal disease student with this problems were excluded in our study .Other studies also suggested that the levels of antioxidants alter in response to any disease or infection process (25). Some studies that evaluate the relationship of TAC and other disease such as periodontitis and diabet mellitus also showed increased TAS in patient subjects similar to dental caries in our study (26-30) .

One study showed a significant relationship between TAC and gender. In our study the results were in agree with that study and females had lower TAC than males. But CA females in comparison to CF females showed higher TAC.

We must be noted here that efficacy of total antioxidant system may be related to many factor such as antioxidants potency and level, amount of FR production, individuals genetic basis, dietary intake, smoking, physical activity ,hormones and stress (22). Therefore in female factors such as hormonal changes and stress can associate with TAC of saliva.

We concluded that there are association between TAC of saliva and dental caries and this may be helpful in caries prevention and progression. Non enzymic and molecular salivary antioxidants such as vitamin E and vitamin C, Uric acid, peroxidase, glutathione peroxidase and the others may be associate with dental caries and other study must be performed to find the relation between the this antioxidants and the effect of using antioxidant in food regimen and dental caries. Also effect of individual antioxidants should be evaluated alone and in association to others antioxidants in dental caries. Investigations about the relation between TAC to gender must be performed future. This study also showed that caries activity can be measured by salivary factors and this is helpful for preventive dentistry.
